# The reliability of the scapular posture and scapular dyskinesis tests in rugby union players

**DOI:** 10.17159/2078-516X/2021/v33i1a11674

**Published:** 2021-10-04

**Authors:** V Singh, K Stokes, G Trewartha, C Mckay

**Affiliations:** 1 University of the West of England, Bristol, School of Health and Social Wellbeing, United Kingdom BS16 1DD; 2 University of Bath. Department for Health, Claverton Down, Bath, United Kingdom of Great Britain and Northern Ireland BA2 7AY

**Keywords:** shoulder, musculoskeletal assessment

## Abstract

**Background:**

Impact forces during rugby can contribute to scapula dysfunction and shoulder pain. Reliable assessment of static and dynamic scapula position is paramount in managing athletes with, and those at risk of developing, shoulder pain.

**Objectives:**

To determine the reliability of static scapular posture (SP), clavicular tilt angle (CTA) and the scapular dyskinesis (SD) assessments by expert and student therapists.

**Methods:**

The study design was an inter-rater and intra-rater reliability study in male university level rugby union players. Four sport rehabilitation students and one experienced physiotherapist evaluated the position of the scapular and clavicle of male university-level rugby union players (inter-rater participants: session 1: n=17, session 2: n=12 and session 3: n=16; (intra-rater participants: n = 12). Participants attended 3 testing sessions, each 1 week apart. Scapular orientation and motion were assessed in five planes of movement and using the Scapular Dyskinesis Test (SDT) respectively. The inter-rater analysis included all participants from each session, while in the intra-rater analysis included only the 12 participants who attended all three testing sessions.

**Results:**

Kappa coefficient values and percentage agreement ratings for students compared to the experienced therapist were: SP=poor to fair (−0.01 to 0.33), (27% – 94%); SDT=slight (0.16; 41%); CTA=fair (0.21; 59%). Test-retest (intra-rater) agreement was fair to moderate (0.22 – 0.44; 69% – 95%), slight (0.12; 47%), and fair (0.39; 77%) for the SP, SDT, and CTA, respectively.

**Conclusion:**

Static and dynamic evaluation of the shoulder by students and an experienced therapist has poor to moderate reliability and should not be used to make clinical decisions based on observation alone.

Synchronised activation of scapulohumeral muscles facilitate optimal shoulder function when tackling in rugby. ^[1]^ Repeated collisions in rugby have the potential to alter this dynamic control, reducing the ability of the shoulder girdle to resist high deceleration forces at the point of impact, resulting in injury. ^[2]^ It is essential that clinicians are able to reliably assess scapular orientation and motion in players at risk of developing shoulder conditions and in those with shoulder pain. ^[3]^

The orientation (anterior tilting and downward rotation) and altered dynamic control of the scapular has been described as being associated with shoulder pain and the risk of injury. ^[4]^ Alterations to the scapular position may be caused by multiple factors, with the large majority related to muscular imbalances and impaired motor control. ^[5]^ Observation of the scapular in resting position can be performed by dividing it into multiple planes of reference, which is consistent with contemporary kinematic analysis. ^[6]^ Separating the evaluation of the scapular into planes of motion has shown moderate inter-rater reliability (0.42) in 15 participants with neck pain. ^[6]^ A recent review demonstrated a lack of consistency in methods of assessing scapular orientation and varied results ranging from 14 out of 26 (54%) to 19 out of 26 (73%) when using the Downs and Black Quality Assessment tool. ^[7]^ In another study the test–retest reliability of this scapular posture rating assessment was investigated by five qualified physical therapists using 50 healthy participants. ^[8]^ The observed agreement between the test and retest ranged from 59% to 87% while the kappa values were inconsistent and showed fair to moderate reliability. The need for subjective judgement may limit the novice rater’s ability to identify impairments. Therefore, O’Leary et al. called for future research to investigate whether differences in the experience levels impacts inter-rater reliability. ^[6]^

The clavicle is part of the shoulder girdle and the clavicle tilt angle (CTA) (angle between the horizontal and long axis of the clavicle) can influence scapular position and be useful in determining scapular orientation. ^[9]^ Multiple impact forces to the clavicle in the rugby tackle may alter the CTA, resulting in abnormal scapular orientation. Clinical evaluation should therefore not overlook the importance of evaluating clavicle position. Goniometric and photographic measurement of the CTA is accessible in clinical practice, though there are no published normative data, which makes the clinical interpretation of the angular measurement unclear.

Scapular malposition and movement impairment is termed scapular dyskinesis (SD) which can be assessed clinically using visual observation including palpation, symptom alteration tests and with more advanced laboratory methods involving three-dimensional motion analysis. ^[10]^ The Scapular Dyskinesis Test (SDT) is a visual observation protocol that has shown moderate inter–rater reliability (0.54) ^[11]^ and concurrent validity has been demonstrated with three-dimensional motion analysis; ^[12]^ however, these studies suffer from diverse test procedures and poor methodological quality. ^[13]^ The primary issue for the modest reliability findings is a lack of consensus of what constitutes a ‘normal’ scapular position, which inevitably influences judgement regarding abnormality. ^[6]^ There is also a paucity of rugby-specific evidence, which offers clinicians limited confidence to use these tests in a population that is exposed to repeated impacts to their shoulders with the propensity to result in injury. ^[14]^

The reliability of clinical tests requires sufficient time for conformity of performance, definitions and evaluations, which it can be argued should be gained with experience and may reflect inconsistent and unreliable ratings by novice raters. Therefore, the purpose of this study was to assess the inter- and intra-rater reliability between an expert therapist and student therapists of static scapular posture, clavicular tilt angle, and scapular dyskinesis tests in rugby union players within a realistic team setting. This research will further inform our understanding about the reliability of these tests when used by student therapists and their contribution in the clinical reasoning process applied in the prevention and management of shoulder complaints in rugby union players.

## Methods

### Participants and setting

An inter- and intra-rater reliability study was conducted with four undergraduate final year sport rehabilitation students and one physiotherapist (15 years of musculoskeletal experience) who independently rated the static scapular posture, clavicular tilt angle and scapular dyskinesis of rugby union players. All students underwent one day of training to familiarise themselves with the testing procedures which were supervised by the physiotherapist prior to the test days. These students also completed 400 supervised clinical hours in the assessment and management of musculoskeletal conditions and also reviewed the literature pertaining to these tests as part of their research training over the past six months.

### Participant characteristics

**Fig. 1:**
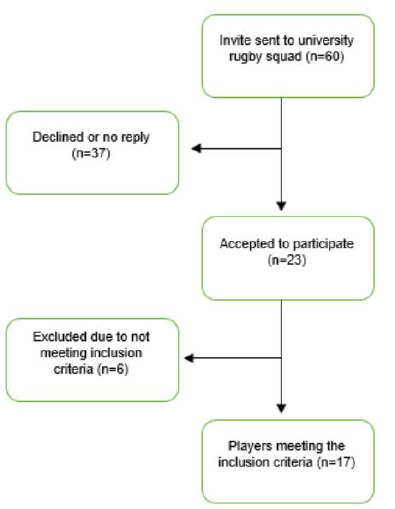
Flowchart of participation through the study

Participants were recruited during the competitive season (September to December 2015) from a squad of 60 players in a university men’s rugby union team (Fig. 1). Participant mean age was 21 years (standard deviation (SD)+/− 1.1 years) and mean weight was 91 kgs (SD +/− 7.9 kgs). Players included in the study reported no current shoulder pain or shoulder injury in the previous six months and had full range of movement in shoulder abduction as screened by the physiotherapist using the painful arc test ^[15]^ before all three testing sessions. Players were excluded if they did not meet these criteria. Players had not trained 18 hours prior to testing. All participants volunteered and provided written informed consent. University of the West of England, Bristol granted ethical approval for this study.

### Procedure

**Fig. 2:**
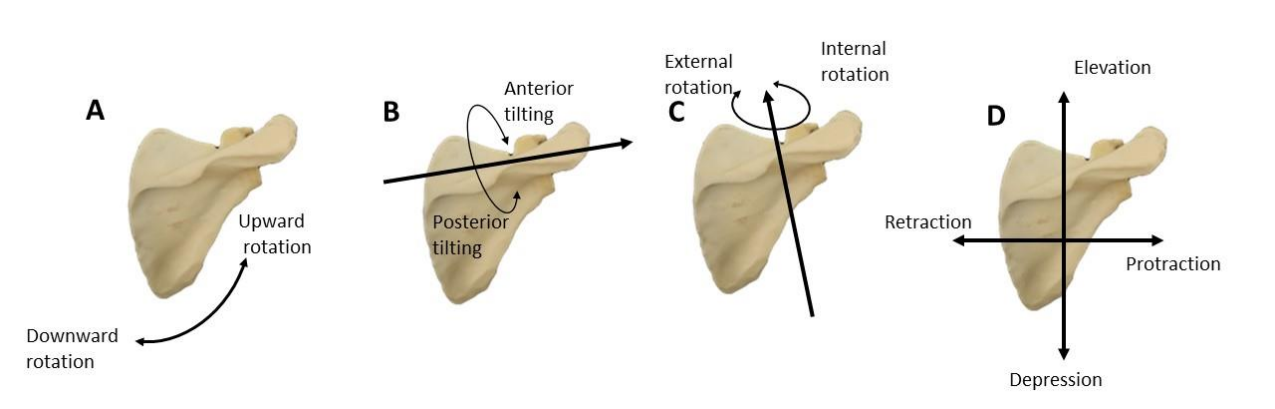
Scapular posture in five planes of movement including the scapular plane (A), sagittal plane (B), transverse plane (C), vertical and horizontal plane (D)

Participants attended three testing sessions (session 1: n=17, session 2: n=12 and session 3 n=16), each one week apart. The inter-rater analysis included all participants from each session, while in the intra-rater analysis included only the 12 participants who attended all three testing sessions. Each participant was randomly allocated to a rater upon arrival for testing in the sports changing room. The following tests were carried out on all three testing days: static scapular orientation, CTA and the SDT. Data collection was conducted at 17h00 before training which meant that raters had a short period in which to perform the assessments. Each player’s profile was completed using a standardised baseline questionnaire that included a randomly generated participant number, date of birth and weight. The rater observed the participant’s scapular posture posteriorly by allocating one of three ordinal ratings in five planes of movement (Fig. 2 and Table 1). The rater then observed the participant from the front so that they could determine the CTA. This measure was made by the rater observing whether the acromioclavicular joint was lower than, level with, or higher than the sternoclavicular joint. All ratings were made with participants standing in the anatomical position. The shoulder’s physical assessments were carried out unilaterally for both arms with the participants barefoot, topless and wearing rugby shorts.

To ensure that all raters observed the same repetitions of the dynamic movement during the SDT, the test was video recorded by a rater at all three testing sessions and the video was viewed and evaluated by all raters the next day. This dynamic test using dumbell hand weights, was done according to the procedure described by McClure et al. (2009) using a video camera capturing a posterior view. The rating of the quality of movement for this test was scored independently for each arm and also independently rated by each rater using the operational definition for the scapular dyskinesis test described in Table 2.

### Statistical analysis

Inter-rater agreement was evaluated with weighted kappa and interpreted using the agreement measures for categorical data. ^[16]^ Each student therapist’s (rater 1 to 4) rating was compared to that of the experienced therapist (VS, rater 5). Intra-rater agreement was evaluated using an unweighted Cohen’s kappa analysis. Each shoulder was treated as independent with pooled data from the left and right ratings analysed for each plane and condition. ^[6]^ Using the kappa coefficient when investigating nominal data can be influenced by the prevalence of responses in each category and bias within the data of paradoxical observations of high exact agreement and low kappa coefficients. ^[17]^ Attempts to correct for this by adjusting the kappa coefficient have been suggested, but are criticised as representing an artificial coefficient when the dataset reflect real-life occurrences. In light of these issues, this study’s approach was to calculate the kappa coefficient and the exact agreement. All analyses were conducted using IBM SPSS Statistics for Windows, Version 24.0. ^[18]^

## Results

### Inter-rater reliability

The reliability for the static scapular posture test ranged from −0.04 to 0.46 (Table 3), which is poor to moderate agreement. Agreement was higher between all raters in the sagittal plane (fair to moderate) than any other plane of movement. Percentage agreement ranged from 18% to 97%.

For the clavicle tilt angle (Table 4), inter-rater reliability ranged from 0.04 to 0.32, which is slight to fair agreement. Percentage agreement ranged from 50% to 64%.

For SDT, inter-rater reliability for abduction ranged from 0.07 to 0.30, which was slight to fair agreement (Table 5). Flexion ranged from −0.02 to 0.18 (slight to fair agreement) and the combined movement ranged from 0.06 to 0.24 (slight to fair agreement). The percentage agreement ranged from 32% to 60%.

### Intra-rater reliability

For scapular posture, intra-rater agreement ranged from 0.00 to 1.00 (Table 6). Agreement of the clavicle tilt angle ranged from 0.02 to 0.65, which is slight to substantial agreement (Table 7).

For SDT, the intra-rater reliability for abduction ranged from −0.17 – 0.55, which was slight to moderate agreement. Flexion ranged from 0.03 – 0.33 (slight to fair agreement), and the combined movement ranged from −0.17 – 0.51 (poor to moderate agreement) (Table 8). The highest agreement was achieved by rater 1 ranging from 0.17 to 0.55 (slight to moderate reliability).

## Discussion

This was the first small field-based study to evaluate inter- and intra-rater reliability between student and experienced therapists for the visual ratings of static scapular posture, clavicular tilt angle and the scapular dyskinesis in asymptomatic rugby union players’ shoulders. Ratings showed a wide range of variability for all tests, yielding generally low reliability. Visual observation of scapular posture varied up to 0.33 (weighted kappa) between different experience levels of raters in this study. The visual rating of the orientation of the scapular using the five planes of motion ^[8]^ as a reference did not have any better utility and did not improve reliability for the experienced or less experienced raters in this study.

The inter-rater agreement was lower (mean kappa 0.16) than reported values in a study that used therapists with different experience levels for the Scapular Dyskinesia Test (0.54). ^[11]^ In our study, raters were provided with test instructions and normal and abnormal motion was described to them; however, they did not train using actual examples of people with abnormal motion, which is considered inherently limiting. ^[11]^ The examples of abnormal motion used in training seem to be an important component, as does the length of training provided for raters. This was apparent in a recent study on 162 elite adolescent handball players which used two physiotherapy students to evaluate the inter-rater reliability for scapular control. ^[19]^ The raters in their study underwent two hours of training followed by two pilot testing sessions prior to data collection which involved 20 physiotherapy students in the first pilot and 45 youth handball players in the second. Evidently their raters received significantly more training than the raters in our study, which may have been a factor in their greater k value (0.67 to 0.84) than in our study. Routine practice of this clinical test is recommended for clinicians to reduce measurement errors.

Agreement for the SDT observed in this study was also lower than that found by novice raters (kappa=0.59) in a study that was published after our study was conducted. ^[20]^ That study was conducted on 40 patients with subacromial impingement and only used two novice raters. They concluded that there were wide confidence intervals with fair limits of agreement (0.38) when using the Landis and Koch ^[16]^ threshold and relatively large differences between the two novice raters for both inter- and intra-rater reliability. A large range in the upper and lower limits for results in that study was also found in our study, which indicates that the SDT is classified and interpreted differently by raters with different experience levels. Using the operational definition in the SDT requires the rater to detect subtle variations of a number of types of dyskinesis which may not be easy to consider simultaneously. The determinants for the thresholds of dyskinesis are not clearly defined and equally may not be obviously apparent. For example, judging when the movement is premature or how much elevation or protraction of the scapula is considered excessive. Unless there is an obvious abnormality present, the likelihood of detecting a subtle abnormality may be low between raters or between time points. The study on elite adolescent handball players, previously mentioned, ^[19]^ used a modified version of the SDT test than that which was described by McClure et al. (2009). The researchers modified the three-option categorisation (normal, subtle or obvious) proposed by McClure et al. (2009) and applied a dichotomised (e.g. absent or present) category instead using normal (normal + subtle dyskinesis) or obvious categories and achieved a greater k value (0.67 to 0.84) than in our study. This has also been argued elsewhere to be a more suitable method to use for research and clinical use. ^[21]^

Combined patterns of dyskinesis exist due to patients’ adaptations, which adds to the complexity of the observation of dyskinesis. ^[22]^ The reliability of other research is not being compared to this study due the different system used. However, it is worth acknowledging that completing fewer than 10 arm elevations/ lowering cycles may be insufficient to elicit a predominant patterns which may have been unclear with the three repetitions used in other research and for that reason might have influenced results in our study which used five repetitions as outlined in the methods from McClure et al. (2009). The importance of being able to reproduce the conditions that impose an increased demand to the shoulder resulting in dysfunctional movement patterns highlighted in the aforementioned elite handball study. ^[19]^ They used a heavier weight dumbbell for male athletes (3kgs) than described by McClure et al. (2009). It was concluded that the choice of the heavier dumbbell may have influenced the greater k value (0.67 to 0.84) than those found in the original study (k= 0.55 to 0.58) ^[11]^ and similarly in our study. It is therefore plausible to recommend that the imposed stress needs to be sufficiently challenging to provoke a movement impairment while taking into consideration the individual’s strength level and sport demand.

Test-retest reliability performed one week apart showed similar fair to moderate reliability (mean range 0.22 – 0.44) as found by other investigators ^[8]^ for scapular posture. High exact agreement was seen with scapular planes with results exceeding 69% agreement which was also similar to previous findings. ^[8]^ A substantial proportion of ratings were different between assessment days, ranging from 0.00 – 1.00 and 33% – 100% for the kappa statistic and percentage agreement, respectively. This wide range of intra-rater reliability coefficients highlight the subjective nature and limitations of this type of clinical evaluation. There are two possible reasons for this disagreement from one testing session to the next: it may be due to rater inconsistency, or an actual difference in scapular posture between sessions. Potential factors that can be considered to have contributed to the variations in the scapular position at subsequent testing sessions could occur from physical activities conducted in between testing days. ^[2]^ Due to inconsistent reliability scores for these tests from one day to the next, their practical use in this sporting environment is brought into question.

Intra-rater reliability of the SDT in this study showed only slight agreement, irrespective of the experience level of the rater. These findings were lower than that found by other investigators sampling inexperienced raters who found substantial to almost perfect agreement in a non-athletic population with shoulder impingement syndrome. ^[20]^ The reasons for these findings are not dissimilar to those already discussed for the variability of the inter-rater agreement. In light of these factors, the low level of reliability for the SDT in this small study, does not provide sufficient support for its use in field – based testing conditions irrespective of the level of experience of the rater.

This study found paradoxical low kappa values but high exact agreement for inter–rater reliability, similar to other investigations. ^[17, 19]^ This was particularly true for the agreement of the scapular in the horizontal (91% – 97%) and vertical plane (88% – 97%), compared to kappa values of 0.00 and −0.02 to 0.00, respectively. Similarly, these findings were evident in the test -retest results in the scapular, vertical and horizontal planes, with kappa values as low as 0.00 while the exact agreement was 100%. These exact agreement values in the horizontal plane are likely due to the position of the medial border of the scapular being more than 5 cmway from the midline and its prominence in the physique of rugby players. Similarly, the position of the scapular in the vertical plane is an indication that the players’ scapulae sit in a neutral position in this plane. In a homogeneous sample in which there is little variability, interpreting the results from the kappa statistic may be misleading.

The first limitation of this study was that the sample size (n=17) was below the general recommendation for obtaining reasonable precision for estimates of reliability that requires at least 50 participants to be recruited to the study, preventing us from drawing firm conclusions regarding the reliability of the shoulder physical examination used in this study. The students could have spent more time training with these assessment methods and pilot testing. The challenging nature of these assessments, testing condition-imposed time constraints and the difference in professional training of physiotherapists and sport rehabilitation students may have had implications on the findings.

## Conclusion

Visual inspection of the static scapular posture, clavicular tilt angle and the scapular dyskinesis between students and an expert physiotherapist had low reliability in this small study. These findings highlight the limitation of this type of clinical evaluation by sports rehabilitation students and warrants that clinicians are aware of their variability. Both tests require further research to determine their validity and clinical utility.

## Figures and Tables

**Table 1:** Criteria for rating scapular posture*

Rating	Criteria
**Scapular plane**	
Upwardly rotated	The inferior angle of the scapular was furthest away from the midline than the superior angle of the scapular.
Neutral	The inferior angle and superior angle of the scapular was equidistant from the midline.
Downwardly rotated	The superior angle of the scapular was furthest away from midline than the inferior angle of the scapular.

**Sagittal plane**	
Anteriorly tilted	The scapular has a prominently raised inferior angle relative to the thorax and the superior angle.
Neutral	The scapular is positioned flat on the thorax with no prominent borders or angles.
Posteriorly tilted	The scapular has a prominently raised superior angle relative to the thorax.

**Transverse plane**	
Internally rotated	The scapular has a prominently raised medial border relative to the thorax.
Neutral	The scapular is rotated forward with no prominence of the medial border of the scapular relative to the thorax.
Externally rotated	The scapular exhibiting minimal or no forward rotation in the transverse plane.

**Vertical plane**	
Elevated	The superior and inferior angle of the scapular superior to T3-4 and T7-9 respectively.
Neutral	The superior and inferior angle of the scapular level with T3-4 and T7-9 respectively.
Depressed	The superior and inferior angle of the scapular inferior to the T3-4 and T7-9 respectively.

**Horizontal plane**	
Protracted	The medial border of the scapular rests more than 5 cm from the midline.
Neutral	The medial border of the scapular rests approximately 5 cm from the midline.
Retracted	The medial border of the scapular rests less than 5 cm from the midline.

* Copyright © 2012 Steven M. McPhail et al. (2014) This is an open access article distributed under the Creative Commons Attribution License, which permits unrestricted use, distribution, and reproduction in any medium, provided the original work is properly cited.

**Table 2:** Scapular Dyskinesis Test: description of operational definitions and rating scale*

**Normal scapulohumeral rhythm**	
Stable scapular with minimal motion during the initial 30° to 60° of shoulder abduction, the scapular then moves smoothly and continuously rotating upward during abduction and smoothly and continuously rotates downward during adduction of the shoulder. No winging is present.	

**Scapular dyskinesis**	
Either one or both of the following abnormalities may be present.	

**Dysrhythmia**	
Scapular motion occurs prematurely or excessive elevation or protraction, during abduction or adduction of the shoulder the motion is not smooth or stuttering, or rapid downward rotation during adduction.	

**Winging**	
The inferior angle and /or medial border of the scapular posteriorly displaced away from the thorax.	

**Rating scale (used for flexion and abduction)**	
**Score**	**Description**
1	Normal motion is depicted by no evidence of abnormality
2	Subtle abnormality is mild or questionable, not consistently present
3	Obvious dysrhythmia or winging is striking, clearly apparent, evident on at least 3/5 repetitions

**Rating scale (used for combined flexion and abduction test movements)**	
**Score**	**Description**
1	Normal is both tests rated as normal or 1 motion is rated as normal and the other is subtle abnormality
2	Subtle abnormality is both flexion and abduction is rated as subtle abnormality
3	Obvious abnormality in either flexion or abduction is rated as obvious abnormality

* Permission granted by Journal of Orthopaedic & Sports Physical Therapy, from McClure P, Tate AR, Kareha S, Irwin D, Zlupko E.

A Clinical Method for Identifying Scapular Dyskinesis, Part 1: Reliability. Journal of Athletic Training. 2009;44(2):160–164.

**Table 3:** Inter-rater agreement for scapular posture (weighted kappa values and percentage agreement) and mean agreement*

Plane of movement	Rater 1 versus 5	Rater 2 versus 5	Rater 3 versus 5	Rater 4 versus 5	Mean
**Scapular**	0.07 (34%)	0.04 (24%)	0.08 (30%)	−0.04 (18%)	0.04 (27%)
**Sagittal**	0.22 (62%)	0.44 (78%)	0.21 (68%)	0.46 (73%)	0.33 (53%)
**Transverse**	0.00 (52%)	0.00 (50%)	0.00 (71%)	0.00 (52%)	0.00 (56%)
**Horizontal**	0.00 (93%)	0.00 (97%)	0.00 (92%)	0.00 (91%)	0.00 (93%)
**Vertical**	−0.02 (93%)	0.00 (97%)	0.00 (97%)	−0.02 (88%)	−0.01 (94%)

Data expressed as weighted kappa (Percentage agreement (%)).

* A 0.00 score was calculated for ratings where the values were a constant and indicated perfect agreement.

Raters 1 to 4 are student therapists; rater 5 is an experienced therapist.

**Table 4:** Inter-rater agreement for observation of clavicle tilt angle (Weighted kappa values and percentage agreement)

Rater	Rater 5						

	Weighted kappa	Standard error	z	value	Lower 95% CI	Upper 95% CI	Percentage agreement (%)
**1**	0.19	0.10	1.89	0.06	−0.00	0.38	58
**2**	0.27	0.10	2.55	0.01	0.07	0.47	62
**3**	0.04	0.06	0.67	0.50	−0.08	0.16	50
**4**	0.32	0.10	3.10	0.00	0.13	0.51	64
**Mean**	0.21						59

Raters 1 to 4 are student therapists; rater 5 is an experienced therapist.

**Table 5:** Inter-rater agreement for scapular dyskinesia (Weighted kappa and percentage agreement)

Rater	Rater 5					

	Abduction		Flexion		Combined	

	Weighted kappa (percentage agreement)	Standard error	Weighted kappa (percentage agreement)	Standard error	Weighted kappa (percentage agreement)	Standard error

**1**	0.07 (57%)	0.09	0.16 (47%)	0.08	0.23 (47%)	0.07
**2**	0.08 (58%)	0.08	−0.02 (38%)	0.07	0.11 (43%)	0.05
**3**	0.30 (60%)	0.09	0.18 (48%)	0.08	0.24 (43%)	0.07
**4**	0.24 (43%)	0.09	0.14 (43%)	0.07	0.06 (32%)	0.06
**Mean kappa**	0.17		0.11		0.16	
**Percentage agreement**	60%		44%		41%	

Raters 1 to 4 are student therapists; rater 5 is an experienced therapist.

**Table 6:** Intra-rater agreement of scapular posture (Cohen’s kappa values and percentage agreement) and mean agreement*

Plane of movement	Rater 1			Rater 2			Rater 3			Rater 4			Rater 5			Mean

	D1 vs D2	D2 vs D3	D1 vs D3	D1 vs D2	D2 vs D3	D1 vs D3	D1 vs D2	D2 vs D3	D1 vs D3	D1 vs D2	D2 vs D3	D1 vs D3	D1 vs D2	D2 vs D3	D1 vs D3	

**Scapular**	0.05	0.01	0.05	0.00	0.00	0.00	0.23	0.06	0.10	0.66	0.30	0.25	0.16	0.07	0.75	0.22
	38%	67%	38%	79%	100%	79%	79%	83%	71%	83%	58%	58%	63%	58%	92%	70%

**Sagittal**	0.41	0.05	0.39	0.11	0.50	0.25	0.56	0.46	0.46	0.25	0.58	0.67	0.48	0.44	0.32	0.43
	75%	63%	75%	67%	83%	75%	83%	75%	75%	63%	79%	83%	75%	75%	67%	74%

**Transverse**	0.14	0.42	0.24	0.52	0.78	0.33	0.00	0.09	0.00	0.28	0.43	0.60	0.27	0.25	0.10	0.34
	63%	33%	46%	79%	92%	71%	92%	83%	92%	63%	71%	83%	58%	63%	42%	69%

**Horizontal**	0.11	0.65	0.07	0.00	0.00	0.00	0.48	0.48	1.00	0.46	0.06	0.65	0.00	0.00	0.00	0.44
	79%	96%	83%	100%	100%	100%	92%	92%	100%	92%	88%	96%	100%	100%	100%	95%

**Vertical**	0.00	0.00	0.00	0.00	0.00	0.00	0.56	0.46	0.46	0.14	0.00	0.00	0.48	0.44	0.32	0.24
	88%	100%	88%	100%	100%	100%	100%	100%	100%	75%	88%	79%	96%	100%	96%	94%

Data expressed as Cohen’s kappa (Percentage agreement (%)).

* A 0.00 score was calculated for ratings where the values were a constant and indicated perfect agreement.

Raters 1 to 4 are student therapists; rater 5 is an experienced therapist. D1, Day 1; D2, Day 2; D3, Day 3.

**Table 7:** Intra-rater agreement for observation of clavicle tilt angle (Cohen’s Kappa values and percentage agreement)

Rater	Day 1 versus 2	Day 2 versus 3	Day 1 versus 3
**1**	0.52 (83%)	0.02 (54%)	0.05 (54%)
**2**	0.57 (79%)	0.50 (75%)	0.42 (71%)
**3**	0.03 (88%)	0.65 (96%)	0.21 (92%)
**4**	0.48 (79%)	0.49 (75%)	0.39 (71%)
**5**	0.58 (79%)	0.52 (79%)	0.48 (75%)
**Mean**	0.39 (77%)		

Data expressed as Cohen’s kappa (Percentage agreement Raters 1 to 4 are student therapists; rater 5 is an experienced therapist.

**Table 8:** Intra-rater reliability for scapular dyskinesis (Cohen’s kappa values and percentage agreement) and mean agreement

Scapular Dyskinesis	Rater 1			Rater 2			Rater 3			Rater 4			Rater 5			Mean

	D1 vs D2	D2 vs D3	D1 vs D3	D1 vs D2	D2 vs D3	D1 vs D3	D1 vs D2	D2 vs D3	D1 vs D3	D1 vs D2	D2 vs D3	D1 vs D3	D1 vs D2	D2 vs D3	D1 vs D3	

**Abduction**	0.55	0.30	0.18	0.26	0.47	0.19	0.02	0.09	0.06	0.26	0.03	0.29	0.20	0.13	0.01	0.20
	79%	67%	63%	71%	79%	67%	50%	50%	50%	71%	63%	71%	71%	54%	46%	63%

**Flexion**	0.28	0.26	0.17	0.03	0.15	0.13	0.17	0.23	0.23	0.08	0.33	0.05	0.07	0.17	0.03	0.16
	67%	54%	29%	58%	54%	29%	63%	58%	21%	46%	67%	46%	38%	42%	29%	47%

**Combined**	0.51	0.23	0.10	0.23	0.17	0.17	0.00	0.17	−0.02	−0.17	−0.08	0.03	0.07	0.02	0.31	0.12
	75%	50%	42%	67%	58%	58%	42%	50%	25%	42%	42%	42%	42%	17%	58%	47%

Data expressed as Cohen’s kappa (Percentage agreement (%)). Raters 1 to 4 are student therapists; rater 5 is an experienced therapist. D1, Day 1; D2, Day 2; D3, Day 3
